# P-152. Comparison of Target Detection Rates of Multiplex Gastrointestinal PCR Panels: A Systematic Literature Review and Meta-analysis

**DOI:** 10.1093/ofid/ofaf695.378

**Published:** 2026-01-11

**Authors:** Jordan Chase, Darsh Devani, Bridget Manning, Lucas Schulz, Thomas Goss

**Affiliations:** Cepheid, Somerville, MA; BeaconOne Healthcare Partners, Boston, Massachusetts; BeaconOne Healthcare Partners, Boston, Massachusetts; Cepheid, Somerville, MA; BeaconOne Healthcare Partners, Boston, Massachusetts

## Abstract

**Background:**

Multiplex gastrointestinal (GI) panels allow rapid detection of a broad range of targets in patients with suspected infectious gastroenteritis (IG). Larger multiplex panels such as a 22- & 15-target panel (22t, 15t) may also detect (or co-detect) colonizing or non-etiologic organisms. This can increase diagnostic uncertainty, overtreatment and healthcare costs. To evaluate the performance of a more focused approach, we performed a systematic literature review (SLR) and meta-analysis (MA) to estimate detection frequency of an 11-target (11t) multiplex GI panel compared to larger panels.

**Figure 1:**
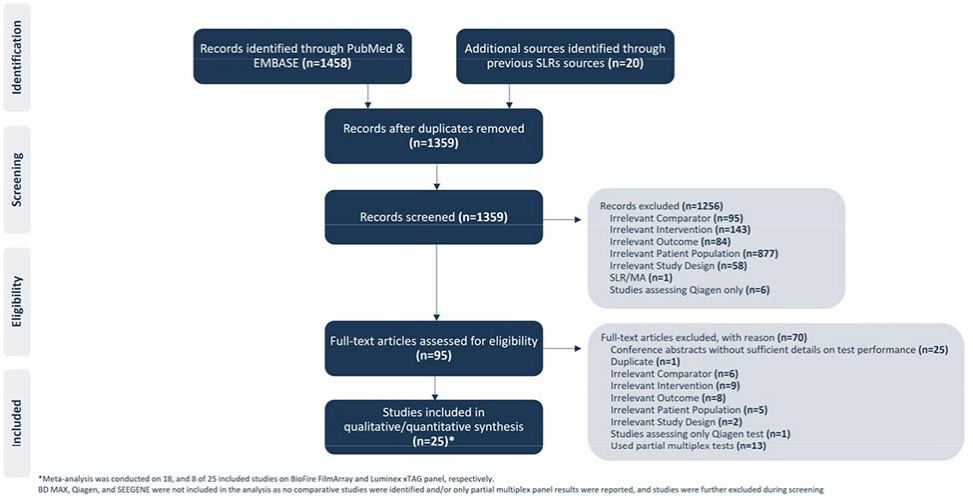
PRISMA Flow Diagram

**Table 1:**
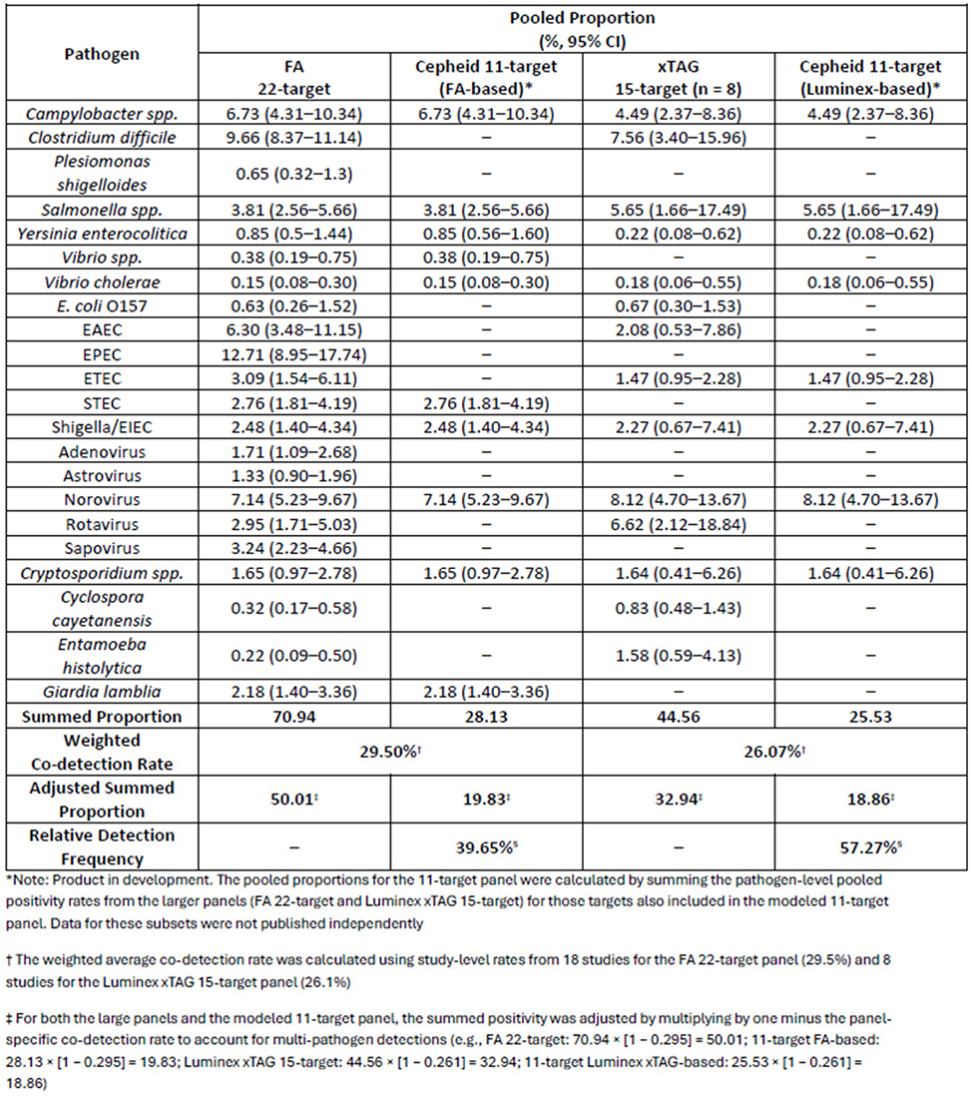
Comparison of Pooled Proportion – BioFire 22-target, Luminex xTAG 15-target & 11-target panel

**Methods:**

We searched PubMed and EMBASE (Jan 2020-Aug 2024) for studies reporting target positivity in suspected IG patients, focusing on 22t & 15t panels. Pooled detection rates were estimated via random-effects meta-analysis. Because no published studies directly evaluated an 11t panel, we estimated its detection using pooled positivity rates for the subsets of targets from larger panels (22t & 15t). Relative detection frequency was then estimated by comparing co-detection-adjusted positivity rates of 11t panel with 22t & 15t panels.

**Table 2:**
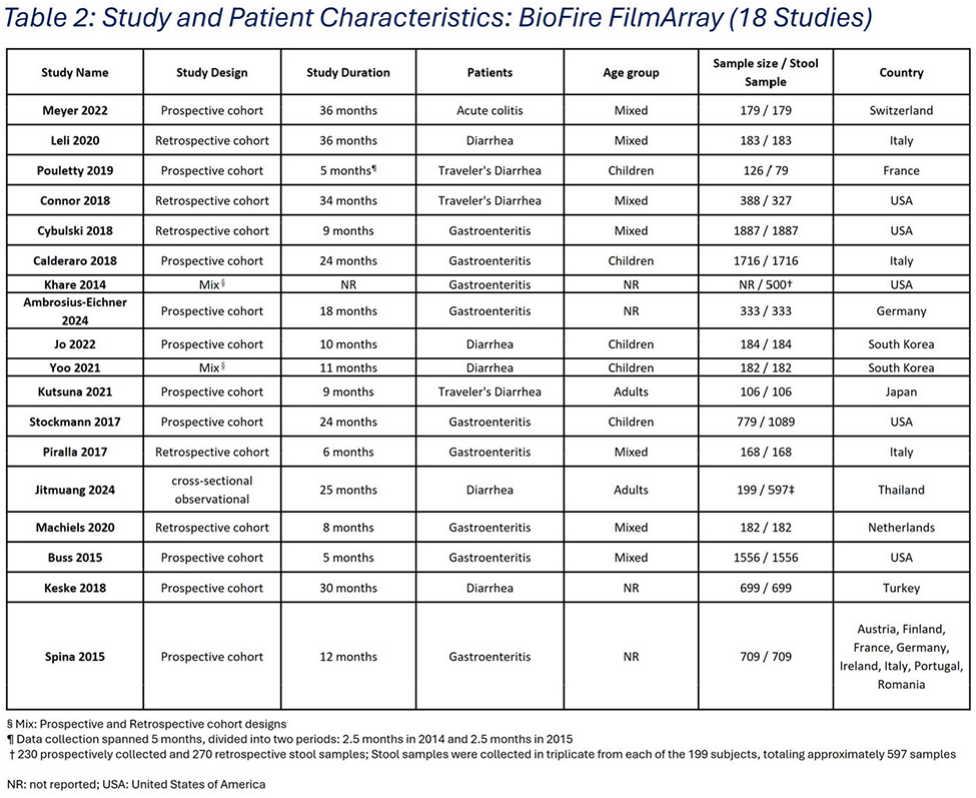
Study and Patient Characteristics: BioFire FilmArray (18 Studies)

**Table 3:**
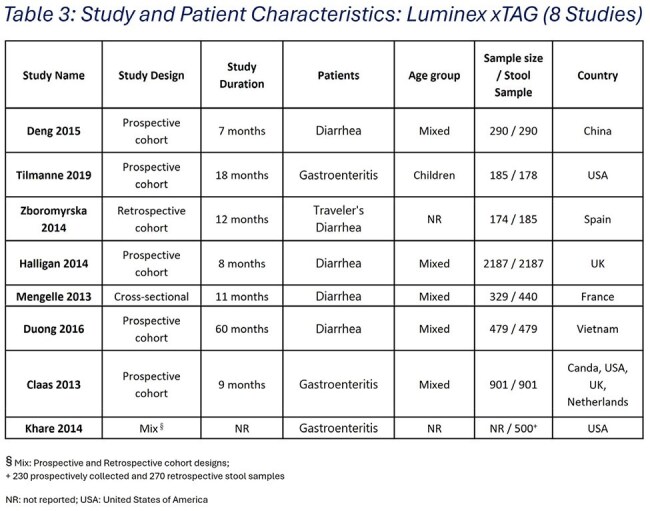
Study and Patient Characteristics: Luminex xTAG (8 Studies)

**Results:**

The SLR and MA included 25 studies, 18 evaluating the 22t & 8 assessing the 15t panels (Figure 1). The adjusted pooled detection for the 22t & 15t panels was 50% & 32.94%, respectively. The 11t panel captured 39.7% & 57.3% of 22t & 15t panels, respectively, suggesting a targeted panel would retain a clinically actionable diagnostic yield by reducing *C. difficile* & certain *E. coli* strains that may be co-detected due to colonization.

**Conclusion:**

Although larger multiplex GI panels yield more positive results, they may contribute to overdiagnosis by detecting non-etiologic or colonizing organisms, including *C. difficile* & *E. coli,* in patients without clinical risk factors or indications for testing those specific targets. An 11t panel captured a meaningful proportion of clinically relevant targets while reducing over- or co-detection of colonizers and pathogens with limited relevance to patient management. These findings align with IDSA & AMP/ASM guidance promoting targeted multiplex testing and support their use improving diagnostic stewardship, limit overtreatment and reduce healthcare costs.

**Disclosures:**

Jordan Chase, BA, Danaher: Stocks/Bonds (Public Company) Darsh Devani, MS, Cepheid: Advisor/Consultant|Cepheid: Grant/Research Support Bridget Manning, MS, Cepheid: Advisor/Consultant|Cepheid: Grant/Research Support Lucas Schulz, PharmD, AbbVie: Advisor/Consultant|Cepheid: Employee Thomas Goss, PharmD, Cepheid: Advisor/Consultant|Cepheid: Grant/Research Support

